# Supplementary material for: FGF21 gene therapy as treatment for obesity and insulin resistance
Source: EMBO Mol Med. 2018 Jul 9;10(8):e8791. doi: 10.15252/emmm.201708791 (PMC6079533; doi:10.15252/emmm.201708791)
Supplement: Supplementary file 1 — Appendix [file EMMM-10-e8791-s001.pdf]

Appendix

FGF21 Gene Therapy as Treatment for Obesity and Insulin Resistance

Veronica Jimenez*, Claudia Jambrina*, Estefania Casana, Victor Sacristan, Sergio Muñoz, Sara Darriba, Jordi Rodó, Cristina Mallol, Miquel Garcia, Xavier León, Sara Marcó, Albert Ribera, Ivet Elias, Alba Casellas, Ignasi Grass, Gemma Elias, Tura Ferré, Sandra Motas, Sylvie Franckhauser, Francisca Mulero, Marc Navarro, Virginia Haurigot, Jesus Ruberte and Fatima Bosch#.

Appendix Figures S1-7.

Appendix Tables S1-S36.

Appendix Figure S1

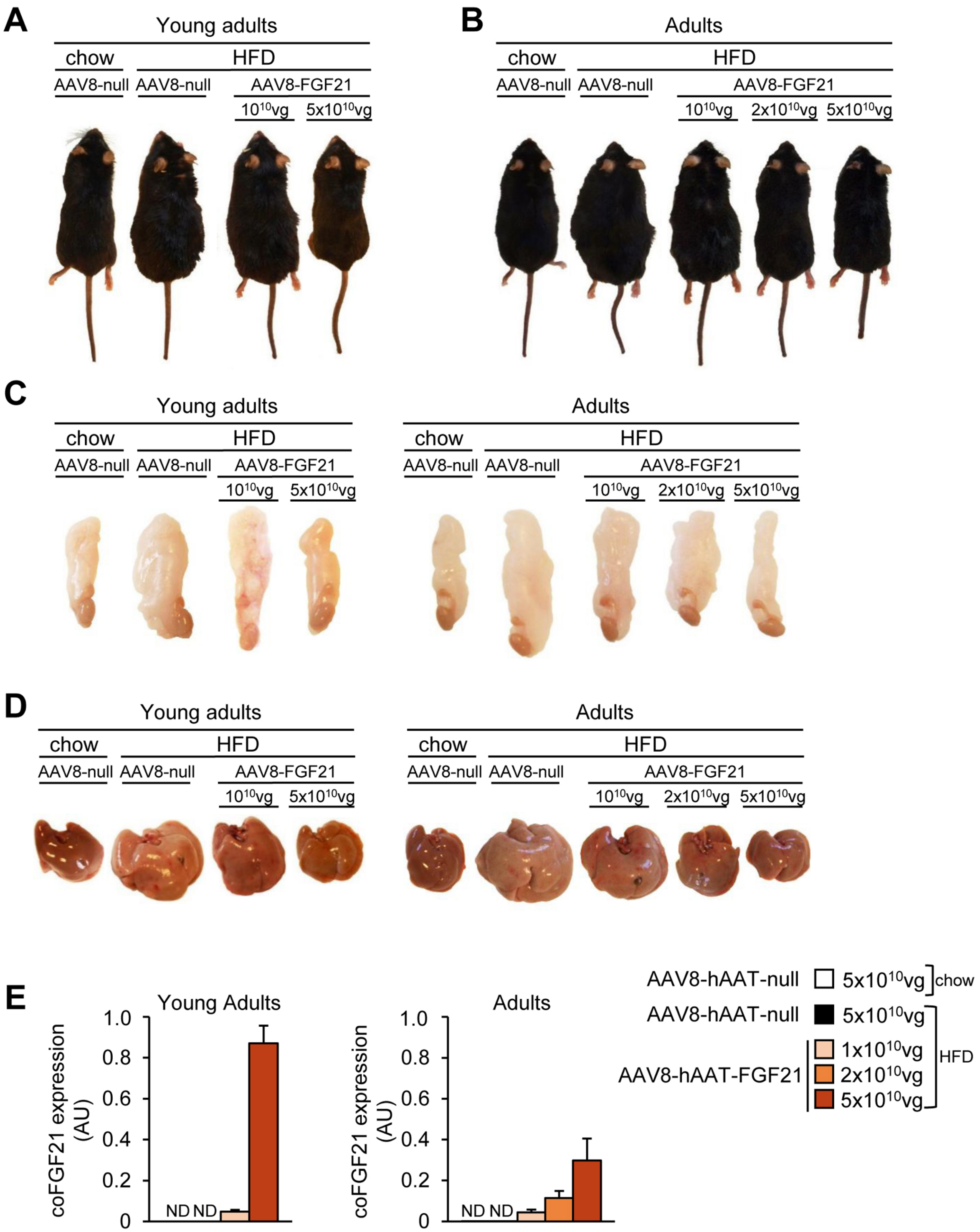

Appendix Figure S1. FGF21 gene transfer to the liver counteracts HFD-induced obesity.

A, B Representative images of animals belonging to all experimental groups of the studies performed in young adults (A) or in adults (B).

C Representative images of the epididymal white adipose (eWAT) pad obtained at sacrifice from animals treated with several doses of AAV8-hAAT-FGF21 as young adults (left) or adults (right).

D Representative images of the liver obtained from animals treated as young adults (left) or adults (right).

E AAV-derived FGF21 expression in the liver of animals treated as young adults or adults. The qPCR was performed with primers that specifically detected the codon-optimized FGF21 (coFGF21) coding sequences.

Data information: All values are expressed as mean ± SEM. In (E) Young adults: AAV8-hAAT-null chow (n=10 animals), AAV8-hAAT-null HFD (n=8), AAV8-hAAT-FGF21 HFD 1x10^{10} vg (n=9) and 5x10^{10} vg (n=8). Adults: AAV8-hAAT-null chow (n=7), AAV8-hAAT-null HFD (n=7), AAV8-hAAT-FGF21 HFD 1x10^{10} vg (n=7), 2x10^{10} vg (n=8) and 5x10^{10} vg (n=7). HFD, High-fat diet.

Appendix Figure S2

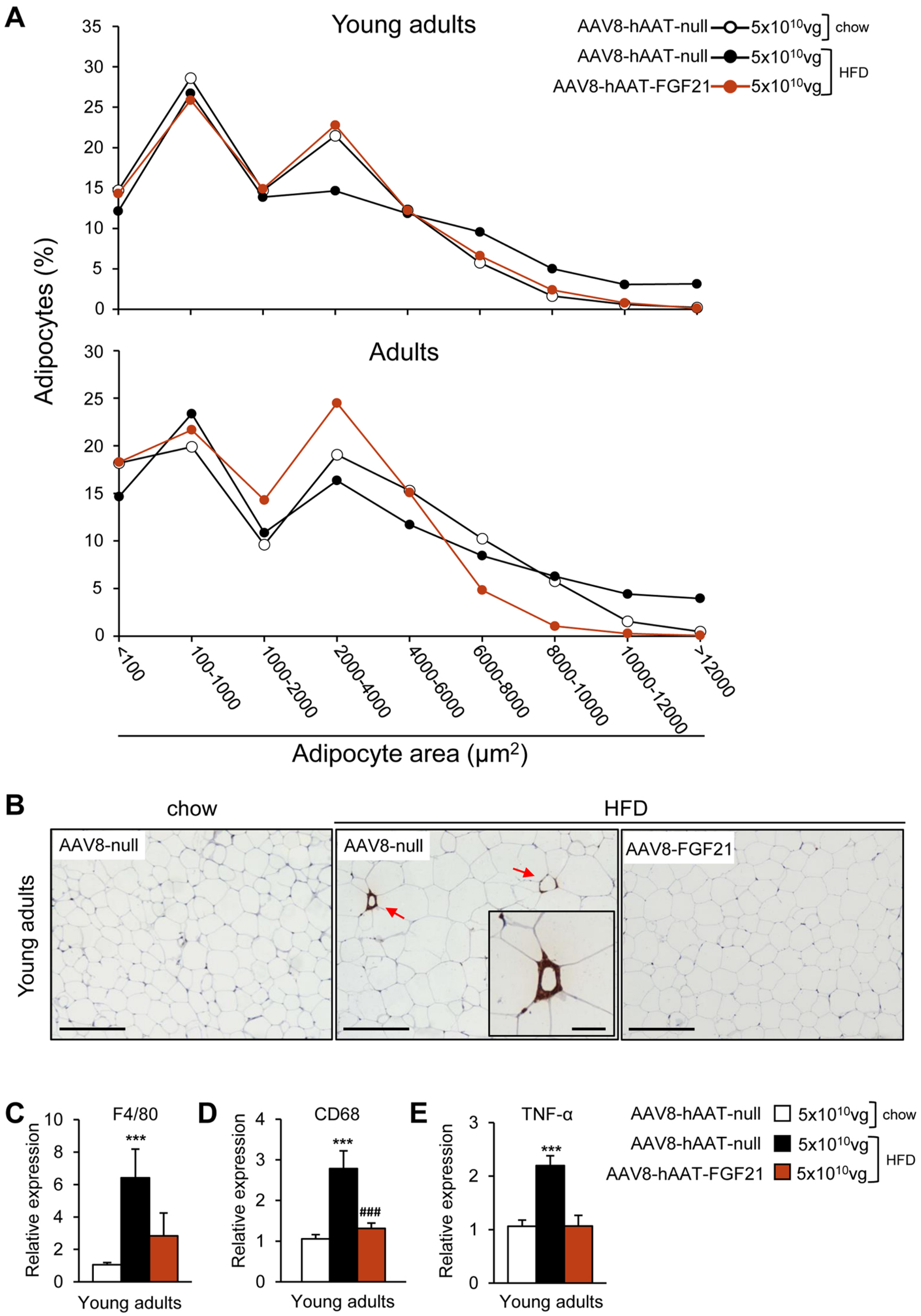

Appendix Figure S2. Adipocyte size and inflammation in AAV8-hAAT-FGF21-treated animals.

A Frequency distribution of adipocyte area in the groups of animals that initiated the chow or HFD feeding and received either AAV8-hAAT-null or $5x10^{10}$ vg/mouse AAV8-hAAT-FGF21 vectors as young adults (top graph) or adults (bottom graph).

B Mac2 immunohistochemistry in eWAT of animals in which the study was initiated as young adults. The crown-like structures formed by infiltrating macrophages in the eWAT of HFD-fed, null-injected mice are indicated by red arrows. Scale bars: 200 μm and 50 μm (inset).

C-E Relative expression by qRT-PCR of the markers of inflammation F4/80 (C), CD68 (D) and TNF-a (E) in the same cohort of animals as in (B).

Data information: All values are expressed as mean ± SEM. In (A) n=4 animals/group. In (C-E) Young adults: AAV8-hAAT-null chow (n=10 animals), AAV8-hAAT-null HFD (n=8), AAV8-hAAT-FGF21 HFD $1x10^{10}$ vg (n=9) and $5x10^{10}$ vg (n=8). Adults: AAV8-hAAT-null chow (n=7), AAV8-hAAT-null HFD (n=7), AAV8-hAAT-FGF21 HFD $1x10^{10}$ vg (n=7), $2x10^{10}$ vg (n=8) and $5x10^{10}$ vg (n=7). In (A-E) data were analyzed by one-way ANOVA with Tukey's post hoc correction. ***P<0.001 versus the chow-fed Null-injected group. ###P<0.001 versus the HFD-fed Null-injected group. HFD, High-fat diet.

Appendix Figure S3

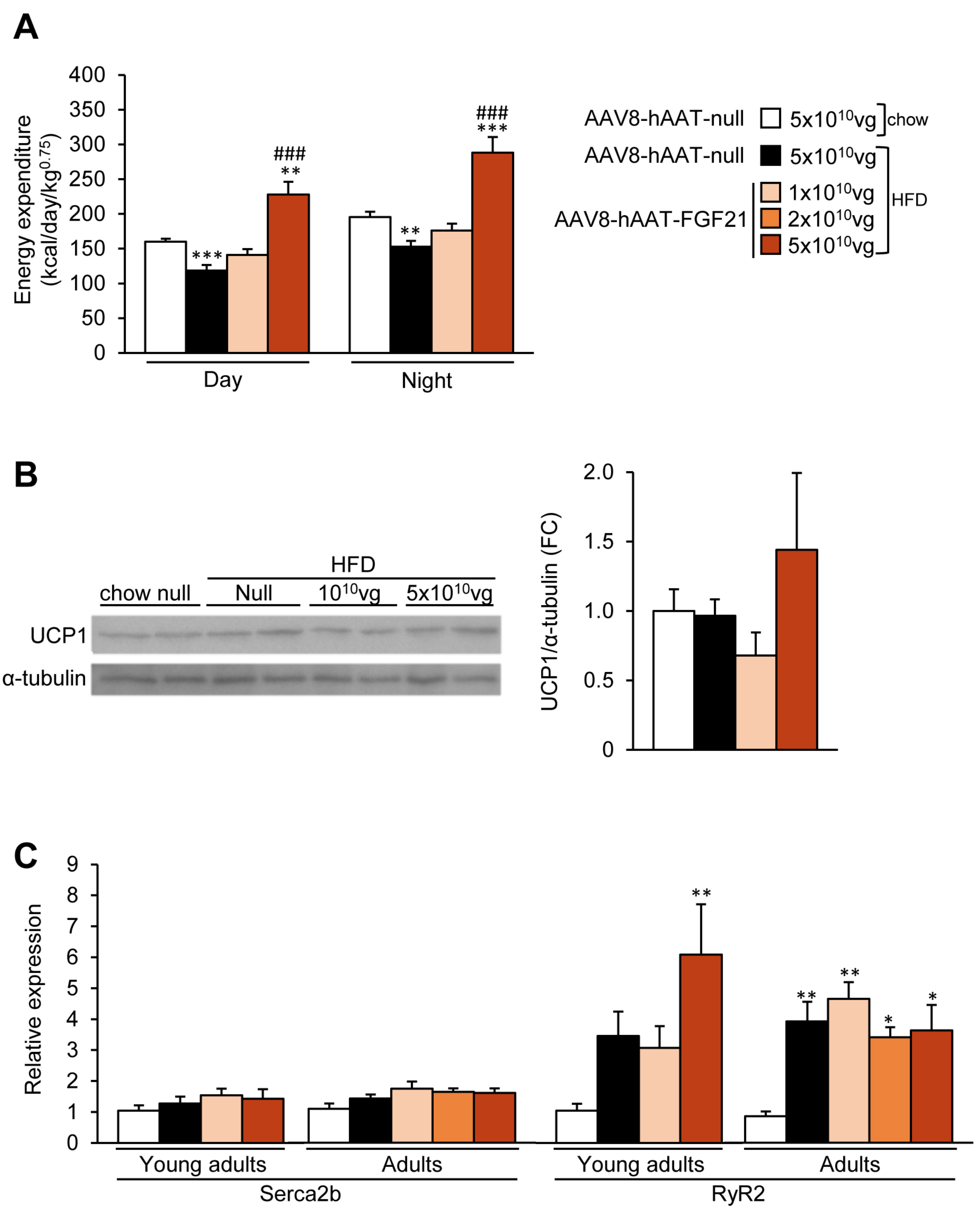

Appendix Figure S3. Energy expenditure 10 months after gene transfer to the liver.

A Energy expenditure was measured 10 months after AAV-hAAT-null or AAV-hAAT-FGF21 vector delivery in the cohort of animals that initiated HFD-feeding at 2 months of age. Data were taken during the light and dark cycles.

B Western-blot analysis of UCP1 content in iWAT from the same cohort of animals. A representative immunoblot is shown (left). The graph shows the densitometric analysis of two different immunoblots (right).

C Relative expression of Serca2b and RyR2 in the iWAT of the groups of animals that initiated the HFD feeding and received FGF21 vectors as young adults or adults.

Data information: All values are expressed as mean ± SEM. In (A, C) Young adults: AAV8-hAAT-null chow (n=10 animals), AAV8-hAAT-null HFD (n=8), AAV8-hAAT-FGF21 HFD $1x10^{10}$ vg (n=9) and $5x10^{10}$ vg (n=8). Adults: AAV8-hAAT-null chow (n=7), AAV8-hAAT-null HFD (n=7), AAV8-hAAT-FGF21 HFD $1x10^{10}$ vg (n=7), $2x10^{10}$ vg (n=8) and $5x10^{10}$ vg (n=7). In (B) n=4 animals/group. In (A-C) data were analyzed by one-way ANOVA with Tukey's post hoc correction. *P<0.05, **P<0.01 and ***P<0.001 versus the chow-fed Null-injected group. ###P<0.001 versus the HFD-fed Null-injected group. HFD, High-fat diet.

Appendix Figure S4

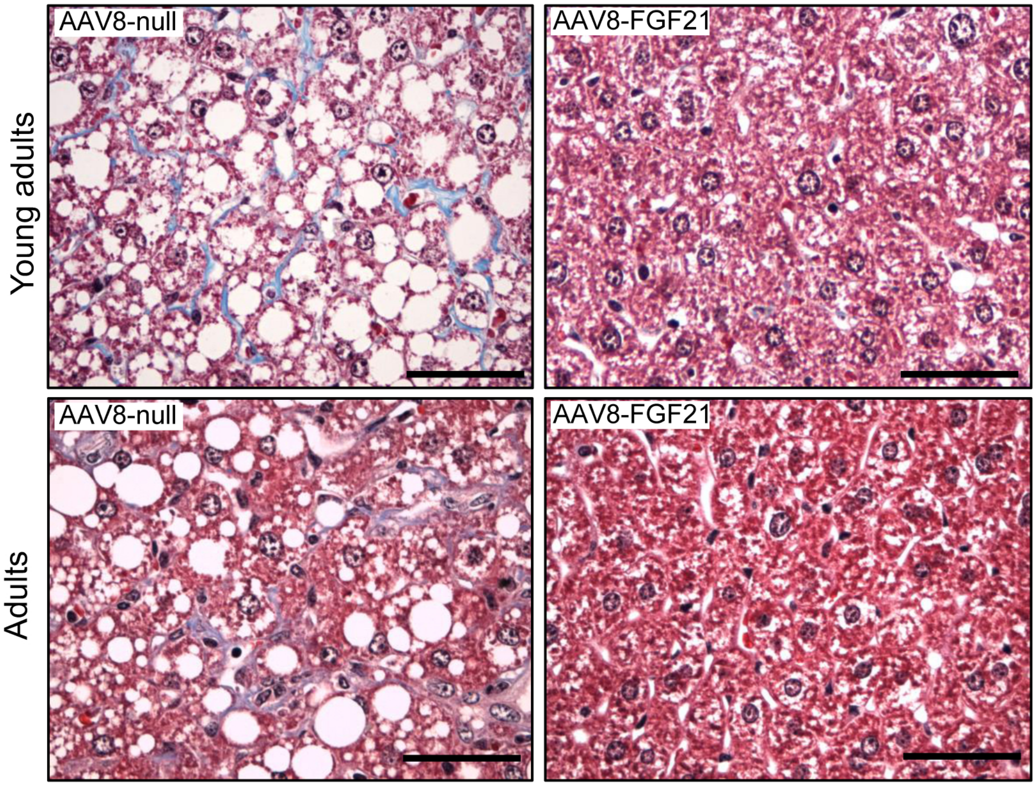

Appendix Figure S4. AAV8-hAAT-FGF21-mediated amelioration of liver fibrosis.

Analysis of hepatic fibrosis through Masson's trichrome staining in animals fed a HFD that received $5x10^{10}$ vg/mouse of either AAV8-hAAT-null or AAV8-hAAT-FGF21 vectors. AAV8-hAAT-FGF21 treatment (right panels) markedly decreased the detection of collagen fibers that were readily detectable (in blue) in animals treated with the null vector (left panels). Scale bars: 50 μm.

Appendix Figure S5

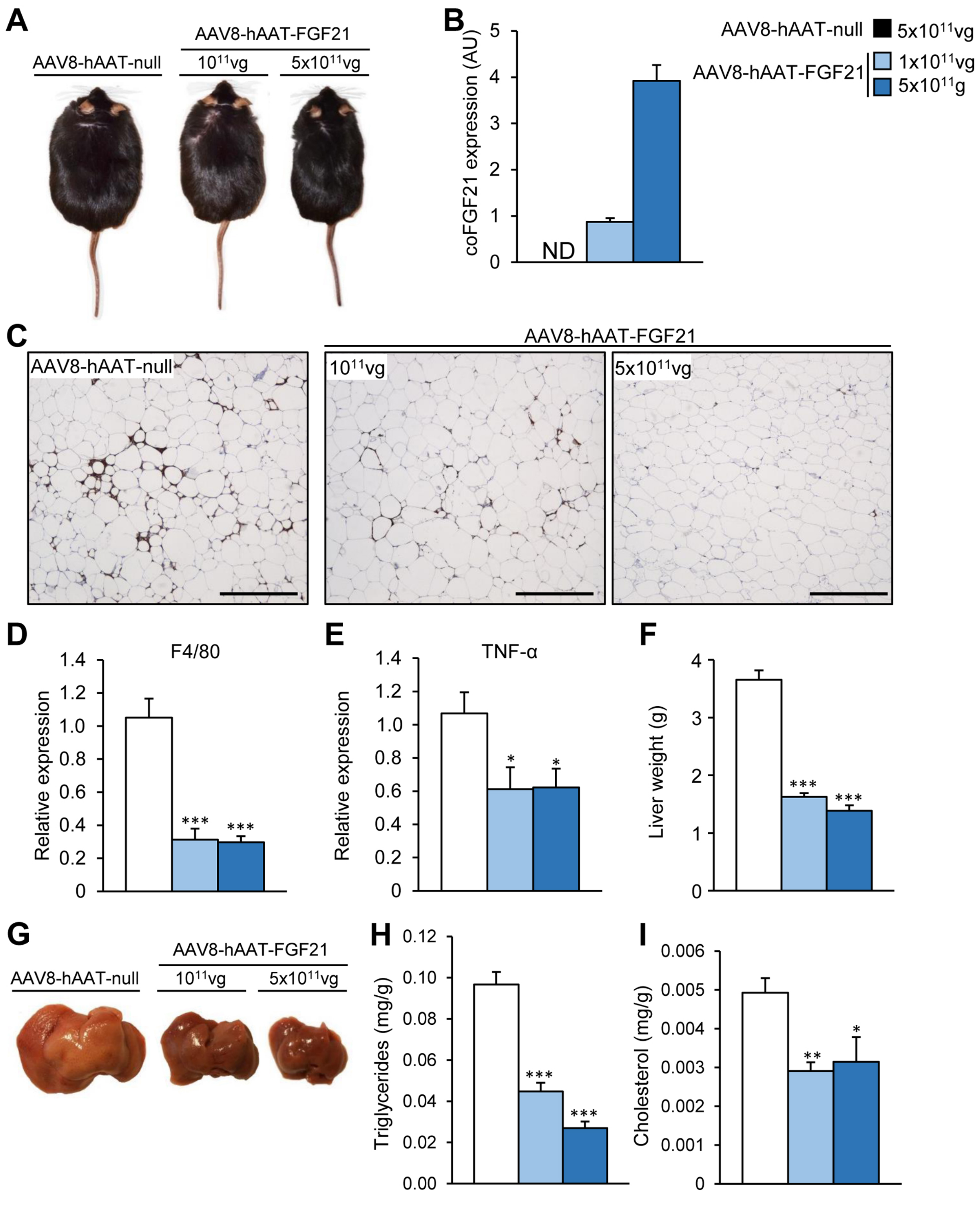

Appendix Figure S5. Effects of FGF21 liver gene transfer on ob/ob mice.

A Representative images of 7-month-old ob/ob animals injected at 2 months of age with either AAV8-hAAT-null vectors or AAV8-hAAT-FGF21 vectors at two different doses ($1x10^{11}$ or $5x10^{11}$ vg/mouse).

B Hepatic expression of AAV-derived FGF21 in the same cohorts as in (A). The qRT-PCR was performed with primers that specifically detected the codon-optimized FGF21 (coFGF21) encoded by the AAV-born expression cassette.

C Immunohistochemistry for the macrophage-specific marker Mac2 in eWAT sections from ob/ob mice that received AAV8-hAAT-FGF21 vectors. Scale bars: 500 µm.

D, E Quantification by qRT-PCR of the expression of the markers of inflammation F4/80 (D) and TNF-a (E) in the same cohorts of mice.

F, G Weight (F) and representative images of the liver (G) obtained from animals belonging to the same experimental groups as in (A).

H, I Hepatic triglyceride and cholesterol content in the fed state in the same cohorts as in (A).

Data information: All values are expressed as mean ± SEM. In (B, D-F, H, I) AAV8-hAAT-null (n=10 animals), AAV8-hAAT-FGF21 $1x10^{11}$ vg (n=10) and $5x10^{11}$ vg (n=9). In (B, D-F, H, I) data were analyzed by one-way ANOVA with Tukey's post hoc correction. *P<0.05, **P<0.01 and ***P<0.001 versus null-injected ob/ob group.

Appendix Figure S6

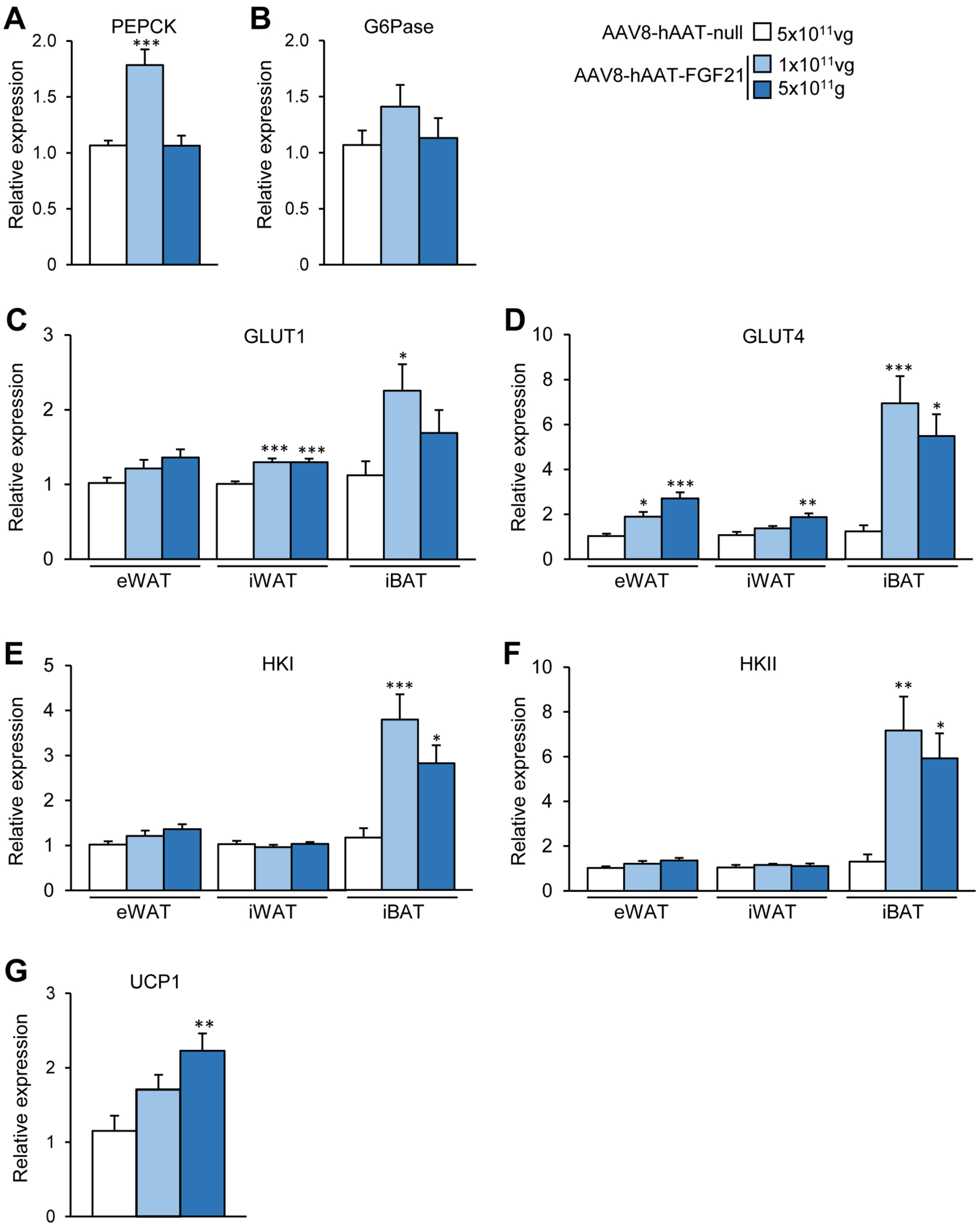

Appendix Figure S6. AAV8-hAAT-FGF21 treatment increases the expression of genes involved in glucose uptake and thermogenesis in adipose tissue of ob/ob mice.

A, B Quantification by qRT-PCR of liver PEPCK (A) and G6Pase (B) expression in ob/ob mice injected at 2 months of age with either AAV8-hAAT-null vectors or AAV8-hAAT-FGF21 vectors.

C-F Quantification by qRT-PCR of GLUT1 (C), GLUT4 (D), HKI (E) and HKII (F) expression in eWAT, iWAT and iBAT in the same animals as in (A).

G Relative expression of UCP1 in iBAT in the same cohorts as in (A).

Data information: All values are expressed as mean ± SEM. In (A-G) AAV8-hAAT-null (n=10 animals), AAV8-hAAT-FGF21 $1x10^{11}$ vg (n=10) and $5x10^{11}$ vg (n=9). In (A-G) data were analyzed by one-way ANOVA with Tukey's post hoc correction. *P<0.05, **P<0.01 and ***P<0.001 versus null-injected ob/ob group.

Appendix Figure S7

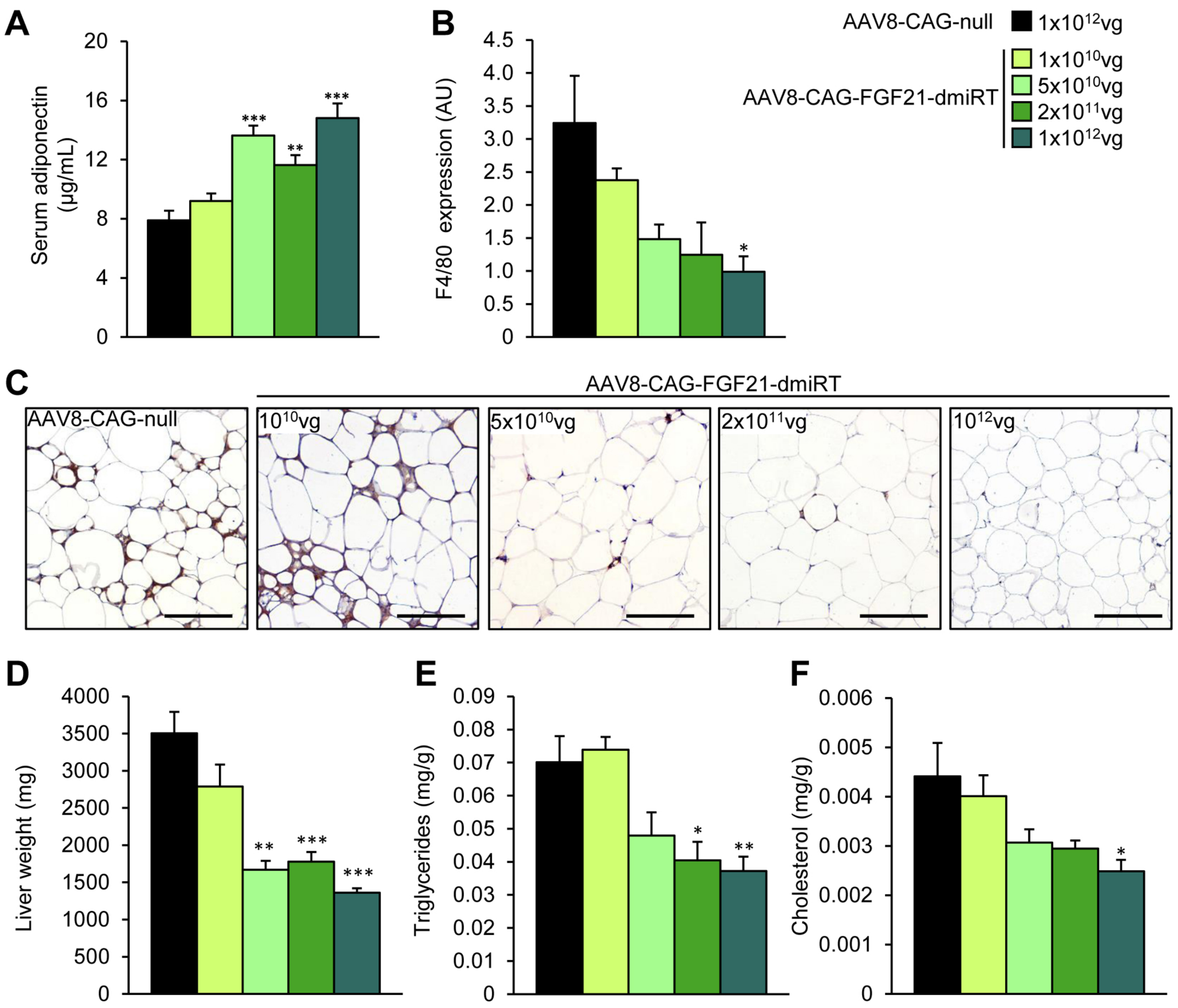

Appendix Figure S7. Impact of FGF21 gene transfer to the eWAT of ob/ob mice.

A Serum adiponectin levels in 25-week-old ob/ob animals injected intra-eWAT at 11 weeks of age with either AAV8-CAG-null vectors or AAV8-CAG-FGF21-dmiRT vectors at 4 different doses ($1x10^{10}$, $5x10^{10}$, $2x10^{11}$, $1x10^{12}$ vg/mouse).

B Quantification by qRT-PCR of the expression of the macrophage marker F4/80 the same groups of animals as in (A).

C Representative images of the immunostaining of eWAT sections from ob/ob mice that received AAV8-CAG-FGF2-dmiRT vectors for the macrophage-specific marker Mac2. Scale bars: 200 µm.

D Weight of the liver in all intra-eWAT treatment groups.

E, F Hepatic triglyceride (E) and cholesterol (F) content in the fed stated in the same cohorts as in (A).

Data information: All values are expressed as mean ± SEM. In (A, B, D-F) AAV8-hAAT-null (n=7 animals), AAV8-hAAT-FGF21 $1x10^{10}$ vg (n=6), $5x10^{10}$ vg (n=4), $2x10^{11}$ vg (n=7) and $1x10^{12}$ vg (n=8). In (A, B, D-F) data were analyzed by one-way ANOVA with Tukey's post hoc correction. *P<0.05, **P<0.01 and ***P<0.001 versus null-injected ob/ob group.

Table S1. Figure 1C significance *P* -values

	**vs chow AAV8-hAAT-null**	**eWAT**	**iWAT**	**rWAT**	**Liver**	**Quadriceps**
**Young adults**	HFD AAV8-hAAT-null	<0.0001	<0.0001	<0.0001	<0.0001	ns
	HFD AAV8-hAAT-FGF21 $1x10^{10}$vg	0.0010	0.0007	0.0001	0.0005	ns
	HFD AAV8-hAAT-FGF21 $5x10^{10}$vg	ns	ns	ns	ns	ns
	**vs HFD AAV8-hAAT-null**	**eWAT**	**iWAT**	**rWAT**	**Liver**	**Quadriceps**
	HFD AAV8-hAAT-FGF21 $1x10^{10}$vg	ns	ns	ns	0.0282	ns
	HFD AAV8-hAAT-FGF21 $5x10^{10}$vg	0.0027	0.0088	0.0004	<0.0001	ns
**Adults**	**vs chow AAV8-hAAT-null**	**eWAT**	**iWAT**	**rWAT**	**Liver**	**Quadriceps**
	HFD AAV8-hAAT-null	0.0068	0.0075	0.0021	<0.0001	ns
	HFD AAV8-hAAT-FGF21 $1x10^{10}$vg	0.0348	0.0039	0.0500	0.0288	ns
	HFD AAV8-hAAT-FGF21 $2x10^{10}$vg	ns	ns	ns	ns	ns
	HFD AAV8-hAAT-FGF21 $5x10^{10}$vg	ns	ns	ns	ns	ns
	**vs HFD AAV8-hAAT-null**	**eWAT**	**iWAT**	**rWAT**	**Liver**	**Quadriceps**
	HFD AAV8-hAAT-FGF21 $1x10^{10}$vg	ns	ns	ns	ns	ns
	HFD AAV8-hAAT-FGF21 $2x10^{10}$vg	ns	ns	ns	0.0024	ns
	HFD AAV8-hAAT-FGF21 $5x10^{10}$vg	0.0028	0.0036	0.0018	<0.0001	ns

ns, non-significant

Table S2. Figure 1D significance *P* -values

	**vs chow AAV8-hAAT-null**	**6 months**	**12 months**
**Young adults**	HFD AAV8-hAAT-null	<0.0001	0.0034
	HFD AAV8-hAAT-FGF21 $1x10^{10}$vg	<0.0001	0.0018
	HFD AAV8-hAAT-FGF21 $5x10^{10}$vg	<0.0001	<0.0001
	**vs HFD AAV8-hAAT-null**	**6 months**	**12 months**
	HFD AAV8-hAAT-FGF21 $1x10^{10}$vg	0.0013	ns
	HFD AAV8-hAAT-FGF21 $5x10^{10}$vg	<0.0001	<0.0001
**Adults**	**vs chow AAV8-hAAT-null**	**3 months**	**8 months**
	HFD AAV8-hAAT-null	0.0002	ns
	HFD AAV8-hAAT-FGF21 $1x10^{10}$vg	<0.0001	<0.0001
	HFD AAV8-hAAT-FGF21 $2x10^{10}$vg	<0.0001	0.0004
	HFD AAV8-hAAT-FGF21 $5x10^{10}$vg	<0.0001	<0.0001
	**vs HFD AAV8-hAAT-null**	**3 months**	**8 months**
	HFD AAV8-hAAT-FGF21 $1x10^{10}$vg	0.0002	0.0003
	HFD AAV8-hAAT-FGF21 $2x10^{10}$vg	<0.0001	0.0009
	HFD AAV8-hAAT-FGF21 $5x10^{10}$vg	<0.0001	<0.0001

ns, non-significant

Table S3. Figure 2 significance *P* -values

**Young adults**	**vs chow AAV8-hAAT-null**	**B**	**C**	**D**			
	HFD AAV8-hAAT-null	0.0319	0.0134	<0.0001			
	HFD AAV8-hAAT-FGF21 $1x10^{10}$vg	ns	ns	0.0002			
	HFD AAV8-hAAT-FGF21 $5x10^{10}$vg	ns	ns	ns			
	**vs HFD AAV8-hAAT-null**	**B**	**C**	**D**			
	HFD AAV8-hAAT-FGF21 $1x10^{10}$vg	ns	ns	ns			
	HFD AAV8-hAAT-FGF21 $5x10^{10}$vg	ns	0.0236	0.0007			
**Adults**	**vs chow AAV8-hAAT-null**	**B**	**C**	**D**	**F**	**G**	**H**
	HFD AAV8-hAAT-null	ns	0.0012	0.0003	0.0140	ns	0.0390
	HFD AAV8-hAAT-FGF21 $1x10^{10}$vg	ns	ns	ns	ns	ns	ns
	HFD AAV8-hAAT-FGF21 $2x10^{10}$vg	ns	ns	ns	ns	ns	ns
	HFD AAV8-hAAT-FGF21 $5x10^{10}$vg	0.0220	ns	ns	ns	ns	ns
	**vs HFD AAV8-hAAT-null**	**B**	**C**	**D**	**F**	**G**	**H**
	HFD AAV8-hAAT-FGF21 $1x10^{10}$vg	ns	0.0073	ns	ns	ns	ns
	HFD AAV8-hAAT-FGF21 $2x10^{10}$vg	ns	0.0012	0.0303	ns	ns	ns
	HFD AAV8-hAAT-FGF21 $5x10^{10}$vg	0.0173	0.0013	0.0157	0.0100	0.0320	ns

ns, non-significant

Table S4. Figure 3A significance *P* -values

	vs chow AAV8-hAAT-null	Food intake
Young adults	HFD AAV8-hAAT-null	ns
	HFD AAV8-hAAT-FGF21 $1x10^{10}$vg	0.0033
	HFD AAV8-hAAT-FGF21 $5x10^{10}$vg	0.0495
	**vs HFD AAV8-hAAT-null**	**Food intake**
	HFD AAV8-hAAT-FGF21 $1x10^{10}$vg	ns
	HFD AAV8-hAAT-FGF21 $5x10^{10}$vg	ns
Adults	**vs chow AAV8-hAAT-null**	**Food intake**
	HFD AAV8-hAAT-null	ns
	HFD AAV8-hAAT-FGF21 $1x10^{10}$vg	<0.0001
	HFD AAV8-hAAT-FGF21 $2x10^{10}$vg	0.0004
	HFD AAV8-hAAT-FGF21 $5x10^{10}$vg	<0.0001
	**vs HFD AAV8-hAAT-null**	**Food intake**
	HFD AAV8-hAAT-FGF21 $1x10^{10}$vg	<0.0001
	HFD AAV8-hAAT-FGF21 $2x10^{10}$vg	0.0009
	HFD AAV8-hAAT-FGF21 $5x10^{10}$vg	<0.0001

ns, non-significant

Table S5. Figure 3B significance *P* -values

	vs chow AAV8-hAAT-null	Day	Night
Young adults	HFD AAV8-hAAT-null	<0.0001	<0.0001
	HFD AAV8-hAAT-FGF21 $1x10^{10}$vg	ns	ns
	HFD AAV8-hAAT-FGF21 $5x10^{10}$vg	<0.0001	<0.0001
	**vs HFD AAV8-hAAT-null**	**Day**	**Night**
	HFD AAV8-hAAT-FGF21 $1x10^{10}$vg	0.0341	0.0220
	HFD AAV8-hAAT-FGF21 $5x10^{10}$vg	<0.0001	<0.0001
Adults	**vs chow AAV8-hAAT-null**	**Day**	**Night**
	HFD AAV8-hAAT-null	ns	0.0475
	HFD AAV8-hAAT-FGF21 $1x10^{10}$vg	ns	ns
	HFD AAV8-hAAT-FGF21 $2x10^{10}$vg	ns	ns
	HFD AAV8-hAAT-FGF21 $5x10^{10}$vg	<0.0001	<0.0001
	**vs HFD AAV8-hAAT-null**	**Day**	**Night**
	HFD AAV8-hAAT-FGF21 $1x10^{10}$vg	ns	ns
	HFD AAV8-hAAT-FGF21 $2x10^{10}$vg	0.0490	0.0807
	HFD AAV8-hAAT-FGF21 $5x10^{10}$vg	<0.0001	<0.0001

ns, non-significant

Table S6. Figure 3C significance *P* -values

	vs chow AAV8-hAAT-null	Distance	Resting	Slow time	Fast time
Young adults	HFD AAV8-hAAT-null	0.0240	0.0449	0.0397	0.0298
	HFD AAV8-hAAT-FGF21 $1x10^{10}$vg	ns	ns	ns	ns
	HFD AAV8-hAAT-FGF21 $5x10^{10}$vg	ns	ns	ns	ns
	**vs HFD AAV8-hAAT-null**	**Distance**	**Resting**	**Slow time**	**Fast time**
	HFD AAV8-hAAT-FGF21 $1x10^{10}$vg	ns	ns	ns	ns
	HFD AAV8-hAAT-FGF21 $5x10^{10}$vg	0.0492	0.0360	0.0402	0.0132

ns, non-significant

Table S7. Figure 3E significance *P* -values

	vs chow AAV8-hAAT-null	UCP1
Young adults	HFD AAV8-hAAT-null	0.0110
	HFD AAV8-hAAT-FGF21 $1x10^{10}$vg	0.0140
	HFD AAV8-hAAT-FGF21 $5x10^{10}$vg	0.0030
	**vs HFD AAV8-hAAT-null**	**UCP1**
	HFD AAV8-hAAT-FGF21 $1x10^{10}$vg	ns
	HFD AAV8-hAAT-FGF21 $5x10^{10}$vg	0.0440

ns, non-significant

Table S8. Figure 3G significance *P* -values

	vs chow AAV8-hAAT-null	Phospho1
Young adults	HFD AAV8-hAAT-null	ns
	HFD AAV8-hAAT-FGF21 $1x10^{10}$vg	ns
	HFD AAV8-hAAT-FGF21 $5x10^{10}$vg	0.0040
	**vs HFD AAV8-hAAT-null**	**Phospho1**
	HFD AAV8-hAAT-FGF21 $1x10^{10}$vg	ns
	HFD AAV8-hAAT-FGF21 $5x10^{10}$vg	0.0010
Adults	**vs chow AAV8-hAAT-null**	**Phospho1**
	HFD AAV8-hAAT-null	ns
	HFD AAV8-hAAT-FGF21 $1x10^{10}$vg	ns
	HFD AAV8-hAAT-FGF21 $2x10^{10}$vg	ns
	HFD AAV8-hAAT-FGF21 $5x10^{10}$vg	<0.0001
	**vs HFD AAV8-hAAT-null**	**Phospho1**
	HFD AAV8-hAAT-FGF21 $1x10^{10}$vg	ns
	HFD AAV8-hAAT-FGF21 $2x10^{10}$vg	ns
	HFD AAV8-hAAT-FGF21 $5x10^{10}$vg	0.0150

ns, non-significant

Table S9. Figure 4 significance *P* -values

Young adults	**vs chow AAV8-hAAT-null**	**TG**	**Cholesterol**	**Collagen**
	HFD AAV8-hAAT-null	<0.0001	<0.0001	<0.0001
	HFD AAV8-hAAT-FGF21 5x10[10]vg	0.0098	0.0014	0.0120
	**vs HFD AAV8-hAAT-null**	**TG**	**Cholesterol**	**Collagen**
	HFD AAV8-hAAT-FGF21 5x10[10]vg	0.0336	0.0212	0.0350
Adults	**vs chow AAV8-hAAT-null**	**TG**	**Cholesterol**	**Collagen**
	HFD AAV8-hAAT-null	0.0007	<0.0001	0.0070
	HFD AAV8-hAAT-FGF21 5x10[10]vg	ns	0.0200	ns
	**vs HFD AAV8-hAAT-null**	**TG**	**Cholesterol**	**Collagen**
	HFD AAV8-hAAT-FGF21 5x10[10]vg	0.0031	0.0475	0.02

ns, non-significant

Table S10. Figure 5B significance *P* -values

Young adults	**vs chow AAV8-hAAT-null**	**Fasted**	**Fed**
	HFD AAV8-hAAT-null	0.0172	0.0076
	HFD AAV8-hAAT-FGF21 1x10[10]vg	ns	ns
	HFD AAV8-hAAT-FGF21 5x10[10]vg	ns	ns
	**vs HFD AAV8-hAAT-null**	**Fasted**	**Fed**
	HFD AAV8-hAAT-FGF21 1x10[10]vg	ns	ns
	HFD AAV8-hAAT-FGF21 5x10[10]vg	0.0010	0.0025
Adults	**vs chow AAV8-hAAT-null**	**Fasted**	**Fed**
	HFD AAV8-hAAT-null	<0.0001	0.00371564
	HFD AAV8-hAAT-FGF21 1x10[10]vg	0.0001	0.0076
	HFD AAV8-hAAT-FGF21 2x10[10]vg	ns	ns
	HFD AAV8-hAAT-FGF21 5x10[10]vg	0.0213	ns
	**vs HFD AAV8-hAAT-null**	**Fasted**	**Fed**
	HFD AAV8-hAAT-FGF21 1x10[10]vg	0.0498	ns
	HFD AAV8-hAAT-FGF21 2x10[10]vg	0.0038	0.0070
	HFD AAV8-hAAT-FGF21 5x10[10]vg	0.0019	0.0077

ns, non-significant

Table S11. Figure 5C significance *P* -values

Young adults	**vs chow AAV8-hAAT-null**	**β-cell mass**
	HFD AAV8-hAAT-null	0.1200
	HFD AAV8-hAAT-FGF21 1x10[10]vg	ns
	HFD AAV8-hAAT-FGF21 5x10[10]vg	ns
	**vs HFD AAV8-hAAT-null**	**β-cell mass**
	HFD AAV8-hAAT-FGF21 1x10[10]vg	0.0281
	HFD AAV8-hAAT-FGF21 5x10[10]vg	0.0212

ns, non-significant

Table S12. Figure 5D, E significance *P* -values

	**vs chow AAV8-hAAT-null**	**15**	**30**	**45**	**60**	**75**	**90**
**Young adults**	HFD AAV8-hAAT-null	0.0009	0.0002	<0.0001	<0.0001	<0.0001	0.0006
	HFD AAV8-hAAT-FGF21 1x10^{10}vg	0.0199	ns	ns	ns	ns	ns
	HFD AAV8-hAAT-FGF21 5x10^{10}vg	ns	0.0100	0.0004	0.0004	0.0002	0.0100
	**vs HFD AAV8-hAAT-null**	**15**	**30**	**45**	**60**	**75**	**90**
	HFD AAV8-hAAT-FGF21 1x10^{10}vg	ns	0.0100	0.0055	0.0048	0.0025	0.0057
	HFD AAV8-hAAT-FGF21 5x10^{10}vg	0.0004	<0.0001	<0.0001	<0.0001	<0.0001	<0.0001
**Adults**	**vs chow AAV8-hAAT-null**	**15**	**30**	**45**	**60**	**75**	**90**
	HFD AAV8-hAAT-null	0.0001	<0.0001	<0.0001	0.0230	ns	ns
	HFD AAV8-hAAT-FGF21 1x10^{10}vg	ns	ns	ns	ns	ns	ns
	HFD AAV8-hAAT-FGF21 2x10^{10}vg	ns	ns	ns	ns	ns	ns
	HFD AAV8-hAAT-FGF21 5x10^{10}vg	ns	0.0020	0.0010	0.0070	0.0300	
	**vs HFD AAV8-hAAT-null**	**15**	**30**	**45**	**60**	**75**	**90**
	HFD AAV8-hAAT-FGF21 1x10^{10}vg	0.0004	0.0040	0.0100	0.0090	ns	ns
	HFD AAV8-hAAT-FGF21 2x10^{10}vg	<0.0001	<0.0001	<0.0001	0.0005	0.0330	ns
	HFD AAV8-hAAT-FGF21 5x10^{10}vg	<0.0001	<0.0001	<0.0001	<0.0001	<0.0001	0.0010

ns, non-significant

Table S13. Figure 5F significance *P* -values

	**vs chow AAV8-hAAT-null**	**15**	**30**	**60**	**90**
**Young adults**	HFD AAV8-hAAT-null	0.0095	ns	0.0014	0.0012
	HFD AAV8-hAAT-FGF21 1x10^{10}vg	ns	ns	0.0153	ns
	HFD AAV8-hAAT-FGF21 5x10^{10}vg	ns	0.0004	ns	ns
	**vs HFD AAV8-hAAT-null**	**15**	**30**	**60**	**90**
	HFD AAV8-hAAT-FGF21 1x10^{10}vg	ns	ns	ns	ns
	HFD AAV8-hAAT-FGF21 5x10^{10}vg	0.0125	<0.0001	0.0002	0.001

ns, non-significant

Table S14. Figure 5G significance *P* -values

	**vs chow AAV8-hAAT-null**	**0**	**15**	**30**	**90**
**Young adults**	HFD AAV8-hAAT-null	0.0269	0.0009	0.0065	0.0070
	HFD AAV8-hAAT-FGF21 1x10^{10}vg	0.0016	0.0012	0.0010	0.0035
	HFD AAV8-hAAT-FGF21 5x10^{10}vg	ns	ns	ns	ns
	**vs HFD AAV8-hAAT-null**	**0**	**15**	**30**	**90**
	HFD AAV8-hAAT-FGF21 1x10^{10}vg	ns	ns	ns	ns
	HFD AAV8-hAAT-FGF21 5x10^{10}vg	0.0104	0.0034	0.0188	0.0006

ns, non-significant

Table S15. Figure 6C significance *P* -values

vs AAV8-hAAT-null	2 months	5 months
AAV8-hAAT-FGF21 1x10^{11}vg	0.0004	<0.0001
AAV8-hAAT-FGF21 5x10^{11}vg	<0.0001	<0.0001

Table S16. Figure 6E, G, H significance *P* -values

vs AAV8-hAAT-null	Adiponectin	Glucose	Insulin
AAV8-hAAT-FGF21 1x10^{11}vg	0.0459	0.0045	<0.0001
AAV8-hAAT-FGF21 5x10^{11}vg	0.0326	0.0009	<0.0001

Table S17. Figure 6I significance *P* -values

vs AAV8-hAAT-null	15	30	45	60	75	90
AAV8-hAAT-FGF21 1x10^{11}vg	0.0197	0.0004	ns	ns	ns	ns
AAV8-hAAT-FGF21 5x10^{11}vg	0.0082	<0.0001	<0.0001	0.0004	0.0010	0.0163

ns, non-significant

Table S18. Figure 7C, G, H significance *P* -values

vs AAV8-CAG-null	FGF21	Glucose	Insulin
AAV8-CAG-FGF21-dmiRT 1x10^{10}vg	ns	ns	0.0035
AAV8-CAG-FGF21-dmiRT 5x10^{10}vg	<0.0001	0.0480	<0.0001
AAV8-CAG-FGF21-dmiRT 2x10^{11}vg	<0.0001	0.0500	<0.0001
AAV8-CAG-FGF21-dmiRT 1x10^{12}vg	<0.0001	0.0270	<0.0001

ns, non-significant

Table S19. Figure 7I significance *P* -values

vs AAV8-CAG-null	15	30	45	60	75	90
AAV8-CAG-FGF21-dmiRT 1x10^{10}vg	ns	ns	ns	ns	ns	ns
AAV8-CAG-FGF21-dmiRT 5x10^{10}vg	ns	0.0094	0.0021	ns	ns	ns
AAV8-CAG-FGF21-dmiRT 2x10^{11}vg	ns	0.0024	0.0011	0.0184	ns	ns
AAV8-CAG-FGF21-dmiRT 1x10^{12}vg	ns	0.0004	0.0003	0.0009	0.0037	ns

ns, non-significant

Table S20. Figure 8A significance *P* -values

vs AAV1-CMV-null	FGF21
AAV1-CMV-FGF21 $3x10^{11}$vg	0.0081

Table S21. Figure 8D significance *P* -values

vs AAV1-CMV-null	Tibialis	eWAT	iWAT	rWAT	iBAT	Liver
AAV1-CMV-FGF21 $3x10^{11}$vg	0.0320	<0.0001	<0.0001	<0.0001	0.0225	0.00192

Table S22. Figure 8E, G significance *P* -values

vs AAV1-CMV-null	TG	Insulin
AAV1-CMV-FGF21 $3x10^{11}$vg	0.0054	0.0115

Table S23. Figure 8H significance *P* -values

vs AAV1-CMV-null	15	30	45	60	75	90
AAV1-CMV-FGF21 $3x10^{11}$vg	0.0020	0.0410	0.0010	0.0050	0.0040	0.0080

Table S24. Expanded View 2A

**Young adults**	**vs chow AAV8-hAAT-null**	**Glucagon**
	HFD AAV8-hAAT-null	ns
	HFD AAV8-hAAT-FGF21 $1x10^{10}$vg	ns
	HFD AAV8-hAAT-FGF21 $5x10^{10}$vg	ns
	**vs HFD AAV8-hAAT-null**	**Glucagon**
	HFD AAV8-hAAT-FGF21 $1x10^{10}$vg	0.0060
	HFD AAV8-hAAT-FGF21 $5x10^{10}$vg	0.0014

ns, non-significant

Table S25. Expanded View 3P

**Young adults**	**vs chow AAV8-hAAT-null**	**IGFBP1**
	HFD AAV8-hAAT-null	0.0044
	HFD AAV8-hAAT-FGF21 $5x10^{10}$vg	ns
	**vs HFD AAV8-hAAT-null**	**IGFBP1**
	HFD AAV8-hAAT-FGF21 $5x10^{10}$vg	ns
**Adults**	**vs chow AAV8-hAAT-null**	**IGFBP1**
	HFD AAV8-hAAT-null	0.0077
	HFD AAV8-hAAT-FGF21 $5x10^{10}$vg	<0.0001
	**vs HFD AAV8-hAAT-null**	**IGFBP1**
	HFD AAV8-hAAT-FGF21 $5x10^{10}$vg	ns

ns, non-significant

Table S26. Appendix Figure S2 C, D, E

	vs chow AAV8-hAAT-null	F4/80	CD 68	TNF-α
Young adults	HFD AAV8-hAAT-null	<0.0001	0.0005	<0.0001
	HFD AAV8-hAAT-FGF21 $5x10^{10}$vg	ns	0.001	0.008

ns, non-significant

Table S27. Appendix Figure S3 A

	vs chow AAV8-hAAT-null	Day	Night
Young adults	HFD AAV8-hAAT-null	0.0002	0.0025
	HFD AAV8-hAAT-FGF21 $1x10^{10}$vg	ns	ns
	HFD AAV8-hAAT-FGF21 $5x10^{10}$vg	0.0004	0.0003
	**vs HFD AAV8-hAAT-null**	**Day**	**Night**
	HFD AAV8-hAAT-FGF21 $1x10^{10}$vg	ns	ns
	HFD AAV8-hAAT-FGF21 $5x10^{10}$vg	0.0003	0.0002

ns, non-significant

Table S28. Appendix Figure S3 C

	vs chow AAV8-hAAT-null	RyR2
Young adults	HFD AAV8-hAAT-null	ns
	HFD AAV8-hAAT-FGF21 $1x10^{10}$vg	ns
	HFD AAV8-hAAT-FGF21 $5x10^{10}$vg	0.0023
	**vs HFD AAV8-hAAT-null**	**RyR2**
	HFD AAV8-hAAT-FGF21 $1x10^{10}$vg	ns
	HFD AAV8-hAAT-FGF21 $5x10^{10}$vg	ns
Adults	**vs chow AAV8-hAAT-null**	**RyR2**
	HFD AAV8-hAAT-null	0.0021
	HFD AAV8-hAAT-FGF21 $1x10^{10}$vg	0.0017
	HFD AAV8-hAAT-FGF21 $2x10^{10}$vg	0.0193
	HFD AAV8-hAAT-FGF21 $5x10^{10}$vg	0.0112
	**vs HFD AAV8-hAAT-null**	**RyR2**
	HFD AAV8-hAAT-FGF21 $1x10^{10}$vg	ns
	HFD AAV8-hAAT-FGF21 $2x10^{10}$vg	ns
	HFD AAV8-hAAT-FGF21 $5x10^{10}$vg	ns

ns, non-significant

Table S29. Appendix Figure S5 D, E, F H, I

vs AAV8-hAAT-null	F4/80	TNF-α	Liver	TG	Cholesterol
AAV8-hAAT-FGF21 $1x10^{11}$vg	<0.0001	0.0236	<0.0001	<0.0001	0.0002
AAV8-hAAT-FGF21 $5x10^{11}$vg	<0.0001	0.0231	<0.0001	<0.0001	0.0275

Table S30. Appendix Figure S6 A

vs AAV8-hAAT-null	PEPCK
AAV8-hAAT-FGF21 $1x10^{11}$vg	0.0002
AAV8-hAAT-FGF21 $5x10^{11}$vg	ns

ns, non-significant

Table S31. Appendix Figure S6 C

vs AAV8-hAAT-null	eWAT	iWAT	iBAT
AAV8-hAAT-FGF21 $1x10^{11}$vg	ns	0.0002	0.0306
AAV8-hAAT-FGF21 $5x10^{11}$vg	ns	0.0003	ns

ns, non-significant

Table S32. Appendix Figure S6 D

vs AAV8-hAAT-null	eWAT	iWAT	iBAT
AAV8-hAAT-FGF21 $1x10^{11}$vg	0.0203	ns	0.0006
AAV8-hAAT-FGF21 $5x10^{11}$vg	<0.0001	0.0013	0.0112

ns, non-significant

Table S33. Appendix Figure S6 E

vs AAV8-hAAT-null	eWAT	iWAT	iBAT
AAV8-hAAT-FGF21 $1x10^{11}$vg	ns	ns	0.0006
AAV8-hAAT-FGF21 $5x10^{11}$vg	ns	ns	0.0338

ns, non-significant

Table S34. Appendix Figure S6 F

vs AAV8-hAAT-null	eWAT	iWAT	iBAT
AAV8-hAAT-FGF21 $1x10^{11}$vg	ns	ns	0.0034
AAV8-hAAT-FGF21 $5x10^{11}$vg	ns	ns	0.0255

ns, non-significant

Table S35. Appendix Figure S6 G

vs AAV8-hAAT-null	UCP1
AAV8-hAAT-FGF21 $1x10^{11}$vg	ns
AAV8-hAAT-FGF21 $5x10^{11}$vg	0.0035

ns, non-significant

Table S36. Appendix Figure S7 A, D, E, F

vs AAV8-CAG-null	Adiponectin	F4/80	Liver	TG	Cholesterol
AAV8-CAG-FGF21-dmiRT $1x10^{10}$vg	ns	ns	ns	ns	ns
AAV8-CAG-FGF21-dmiRT $5x10^{10}$vg	0.0008	ns	0.0013	ns	ns
AAV8-CAG-FGF21-dmiRT $2x10^{11}$vg	0.0053	ns	0.0008	0.0188	ns
AAV8-CAG-FGF21-dmiRT $1x10^{12}$vg	<0.0001	0.0113	<0.0001	0.0023	0.0129

ns, non-significant
