# Supplementary material for: FGF21 gene therapy as treatment for obesity and insulin resistance
Source: EMBO Mol Med. 2018 Jul 9;10(8):e8791. doi: 10.15252/emmm.201708791 (PMC6079533; doi:10.15252/emmm.201708791)
Supplement: Supplementary file 2 — Expanded View Figures PDF [file EMMM-10-e8791-s002.pdf]

Expanded View Figures

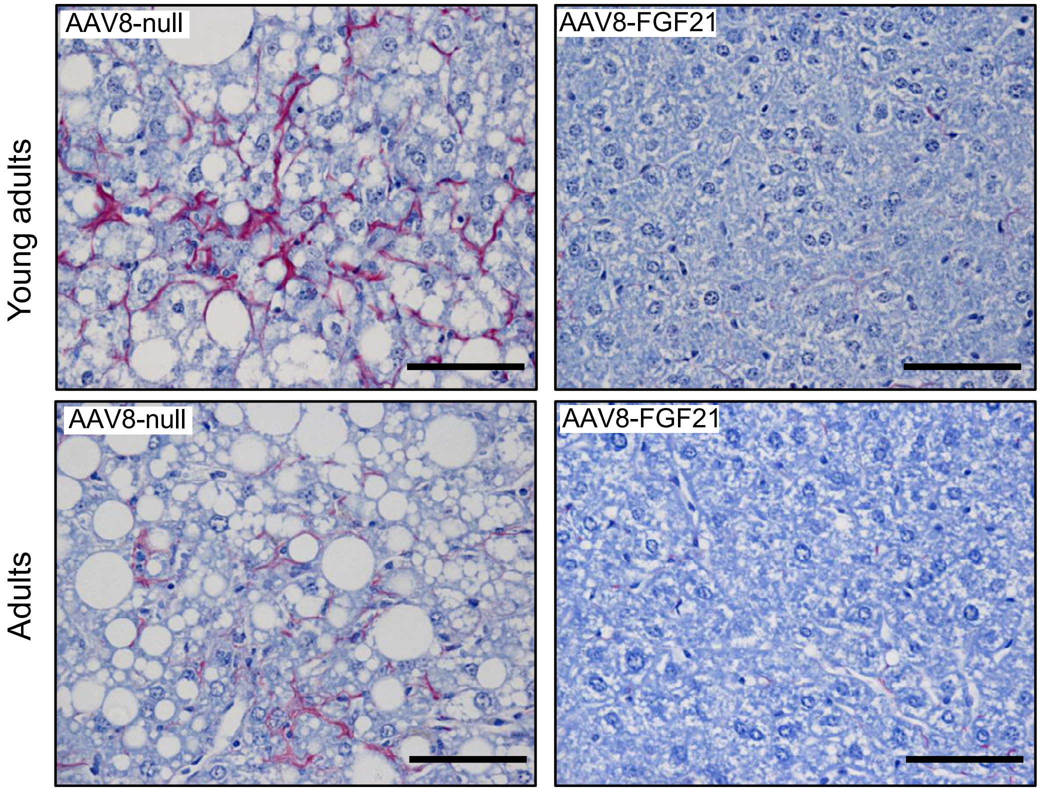

Figure EV1. AAV8-hAAT-FGF21 treatment improves liver fibrosis.

Analysis of hepatic fibrosis through PicroSirius staining in animals fed a HFD that received 5×10^{10} vg/mouse of either AAV8-hAAT-null or AAV8-hAAT-FGF21 vectors. AAV8-hAAT-FGF21 treatment (*right panels*) markedly decreased the detection of collagen fibers that were readily detectable (in red) in animals treated with the null vector (*left panels*). Scale bars: 50 μm.

Figure EV2. AAV8-hAAT-FGF21-mediated reversal of islet hyperplasia.

A Fasted glucagon levels in the group of animals that initiated the HFD feeding and received FGF21 vectors as young adults.
B Representative images of the immunostaining against insulin in pancreas sections from animals that received 5×10^{10} vg/mouse of AAV8-hAAT-FGF21 as adults. Scale bars: 400 μm. Inset scale bars: 100 μm.
C Representative images of the double immunostaining against insulin (in green) and glucagon (in red) in pancreas sections from animals that received 5×10^{10} vg/mouse of AAV8-hAAT-FGF21 as young adults (*upper panel*) or adults (*lower panel*). Scale bars: 100 μm.

Data information: All values are expressed as mean ± SEM. In (A), young adults: AAV8-hAAT-null chow (n = 10 animals), AAV8-hAAT-null HFD (n = 10), AAV8-hAAT-FGF21 HFD 1×10^{10} vg (n = 9), and 5×10^{10} vg (n = 10). In (A), data were analyzed by one-way ANOVA with Tukey's post hoc correction. $^{\#}P < 0.05$ and $^{\#\#}P < 0.01$ versus the HFD-fed null-injected group. HFD, High-fat diet.

▶

© 2018 The Authors

© 2018 The Authors EMBO Molecular Medicine e8791 | 2018 25 of 24

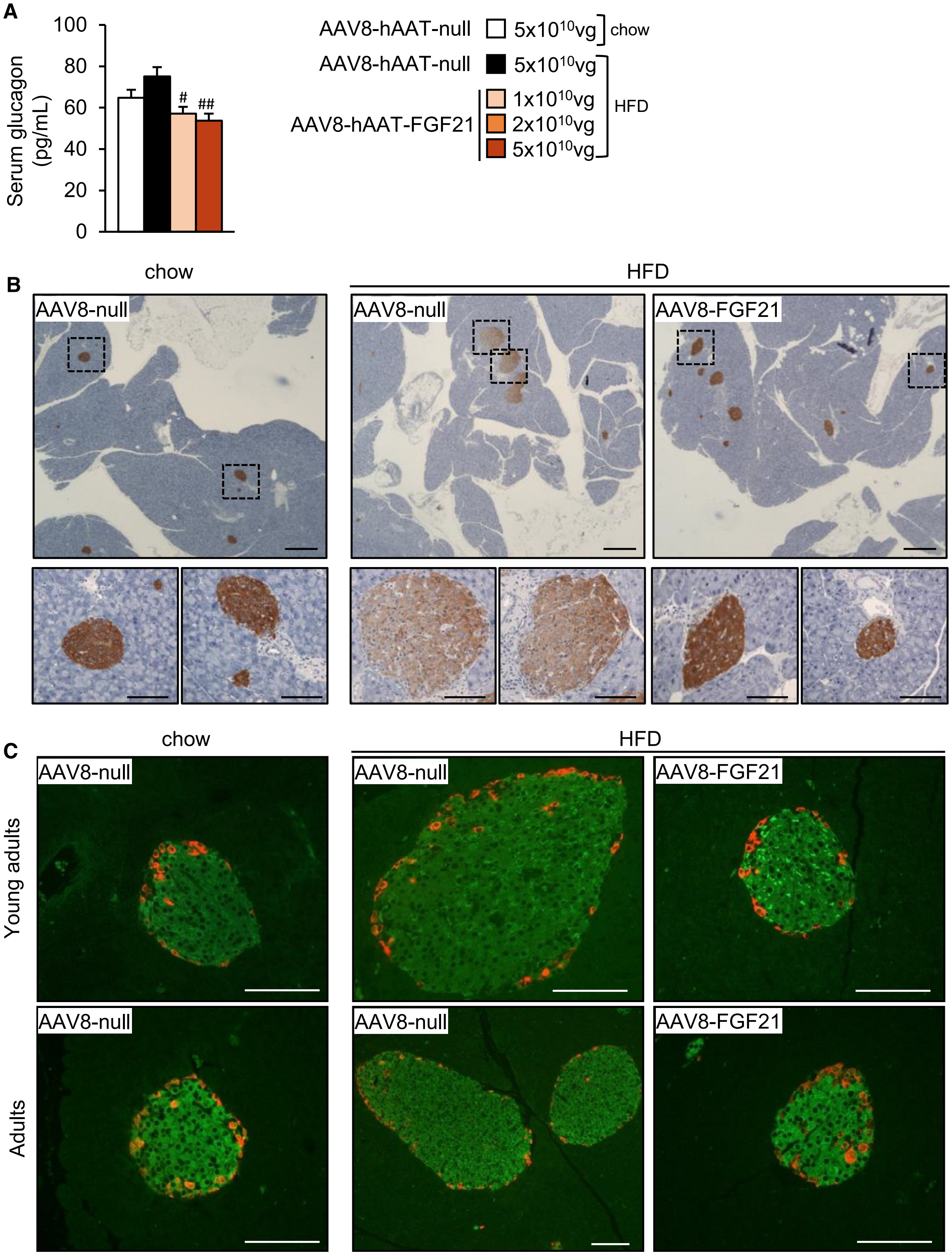

Figure EV2.

 © 2018 The Authors

Figure EV3. No bone abnormalities were observed in AAV8-hAAT-FGF21-treated animals.

The long-term effects of FGF21 gene transfer on bones were studied by comparison of HFD-fed mice treated with the highest dose (5×10^{10} vg/mouse) of FGF21 vectors as young adults or adults with null-injected, chow or HFD-fed animals.

A Total naso-anal length.
B Tibial length.
C–O Micro-computed tomography (μCT) analysis of the epiphysis (C–J) and the diaphysis (K–O) of tibiae obtained at the time of sacrifice, that is, when animals were 18 months of age, from HFD-fed mice administered with either null or FGF21-encoding AAV vectors.
P, Q Circulating IGFBP1 (P) and IGF1 (Q) levels measured by ELISA.

Data information: All data represent the mean ± SEM. In (A, P, Q), Young adults: AAV8-hAAT-null chow (n = 10 animals), AAV8-hAAT-null HFD (n = 8), AAV8-hAAT-FGF21 HFD 1×10^{10} vg (n = 9), and 5×10^{10} vg (n = 8). Adults: AAV8-hAAT-null chow (n = 7), AAV8-hAAT-null HFD (n = 7), AAV8-hAAT-FGF21 HFD 1×10^{10} vg (n = 7), 2×10^{10} vg (n = 8), and 5×10^{10} vg (n = 7). In (B–O), n = 4 animals/group. In (A, B, P, Q), data were analyzed by one-way ANOVA with Tukey's post hoc correction. In (C–O), data were analyzed by unpaired Student's t-test. $^{**}P < 0.01$ and $^{***}P < 0.001$ versus the chow-fed null-injected group. HFD, high-fat diet; BMD, bone mineral density; BMC, bone mineral content; BV, bone volume; BV/TV, bone volume/tissue volume ratio; BS/BV, bone surface/bone volume ratio; Tb.N, trabecular number; Tb.Th, trabecular thickness; Tb.Sp, trabecular separation.

© 2018 The Authors

© 2018 The Authors

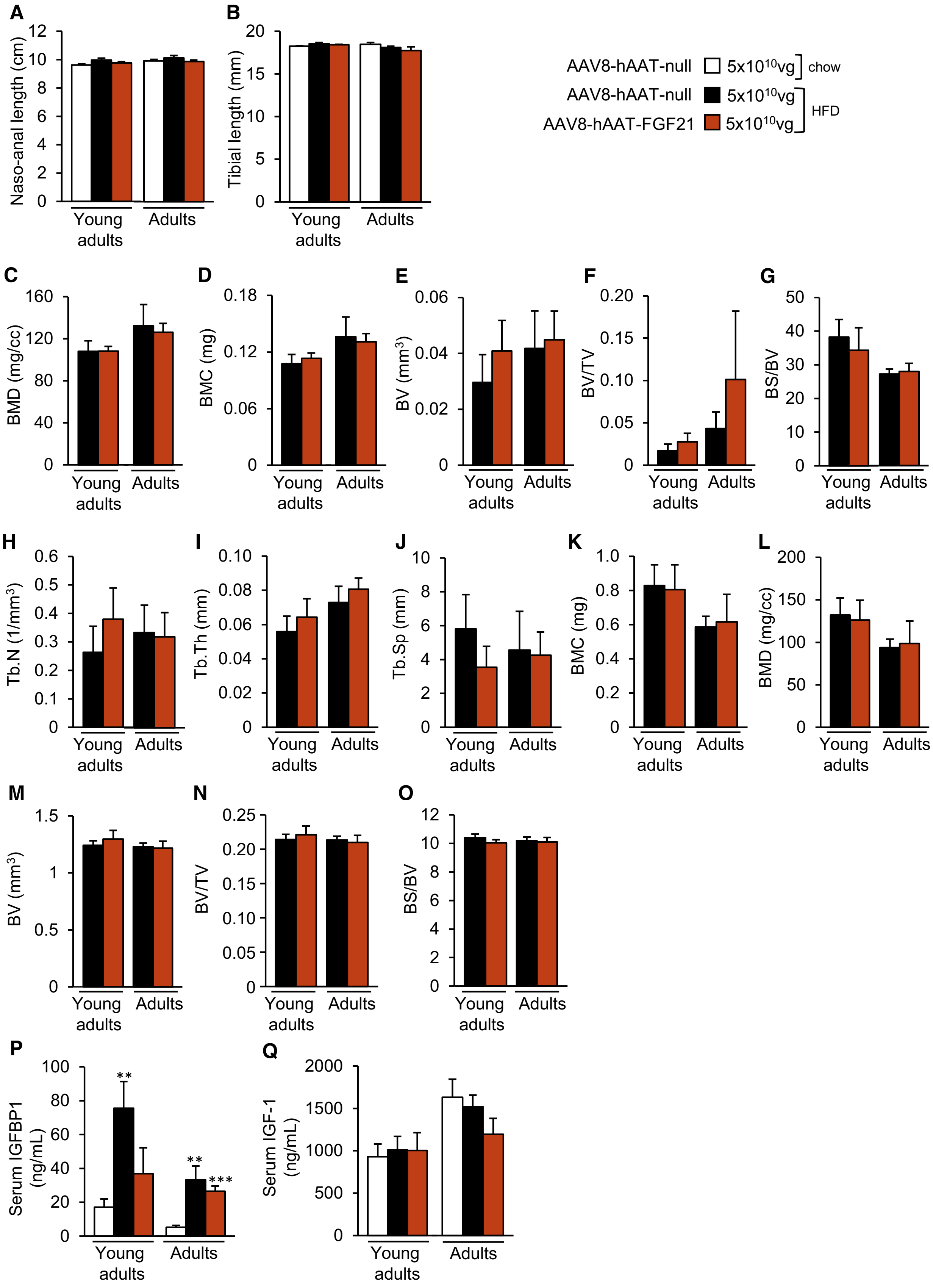

Figure EV3.

© 2018 The Authors
