# Supplementary figures and images for: FGF21 gene therapy as treatment for obesity and insulin resistance
Source: EMBO Mol Med. 2018 Jul 9;10(8):e8791. doi: 10.15252/emmm.201708791 (PMC6079533; doi:10.15252/emmm.201708791)

Source Data Appendix Figure S3B

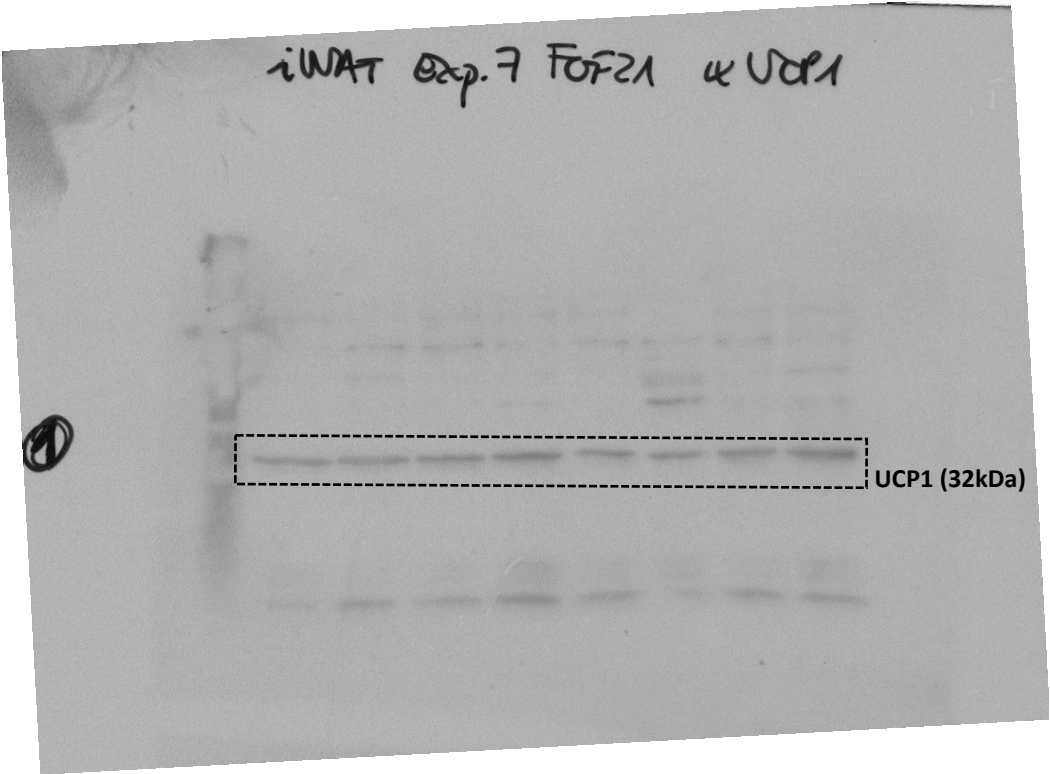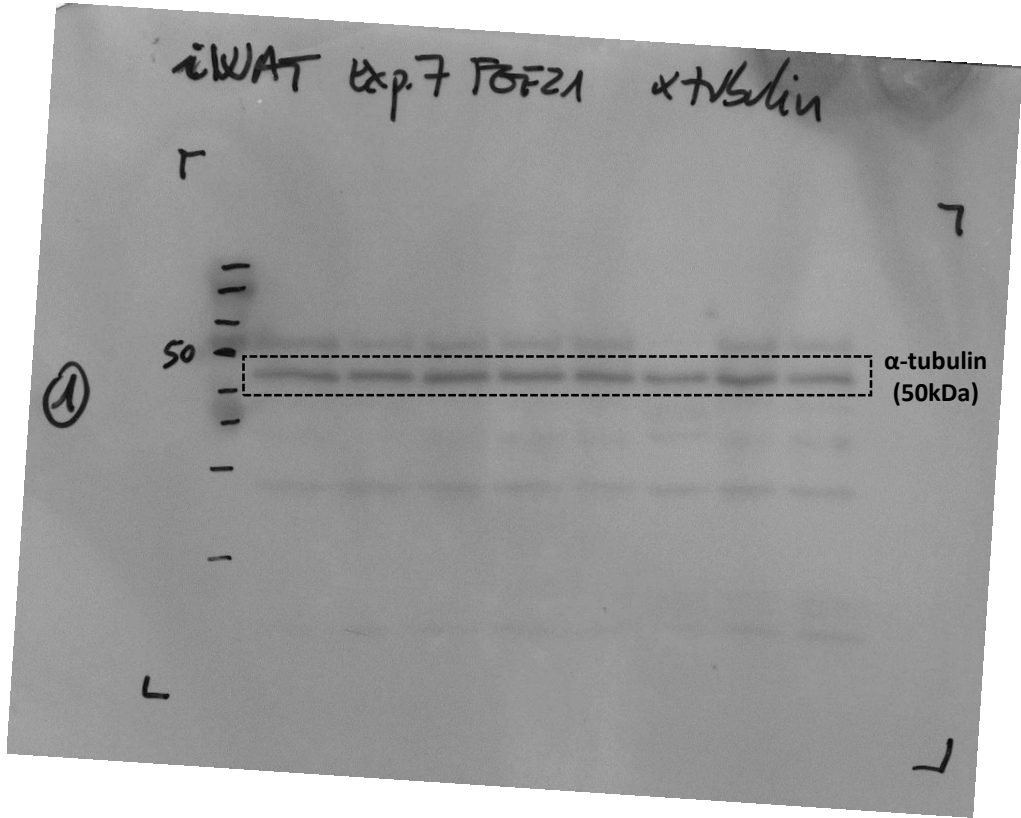

Supplement: Supplementary file 3 — Source Data for Appendix [file EMMM-10-e8791-s004.zip › emmm201708791-sup-0004-SDataAppendixfig3b.pdf]

Source Data Figure 3E

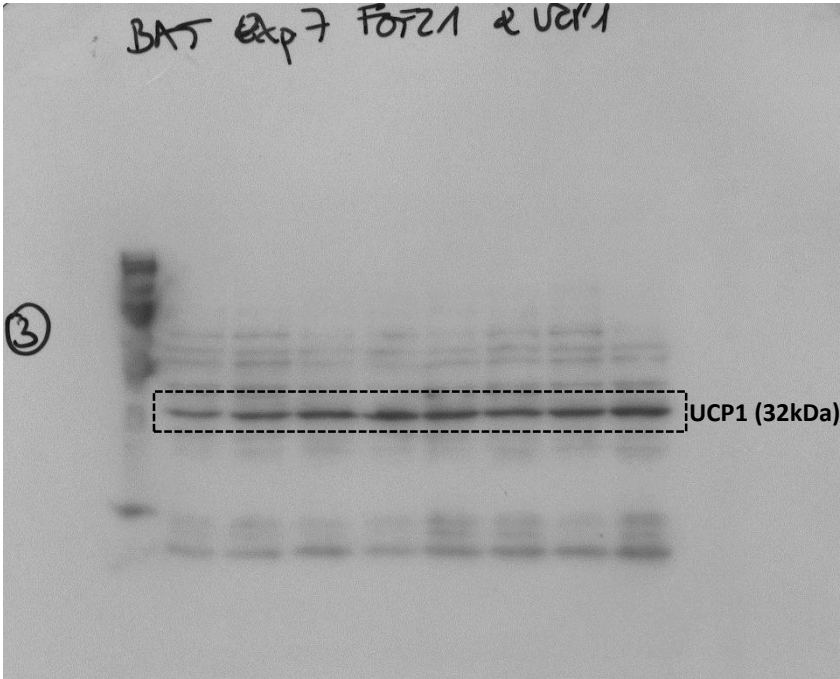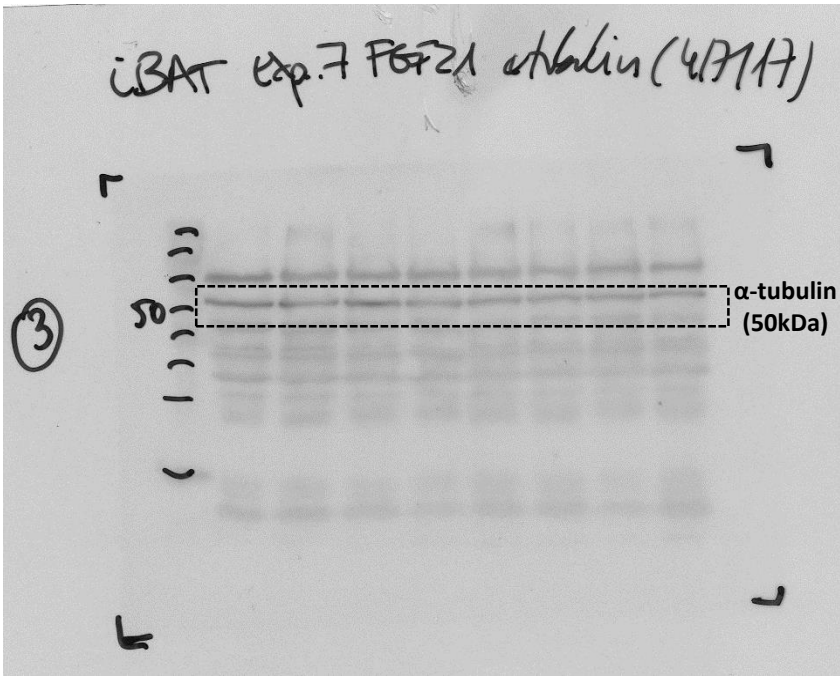

Supplement: Supplementary file 5 — Source Data for Figure 3 [file EMMM-10-e8791-s003.pdf]
